# The Effect of Attitudes Towards Individuals with Sexual Convictions on Professional and Student Risk Judgments

**DOI:** 10.1177/10790632211070799

**Published:** 2022-02-28

**Authors:** Craig A. Harper, Rachel A. Hicks

**Affiliations:** 1 6122 Nottingham Trent University (UK), Nottingham, UK; 2 Nottinghamshire Healthcare NHS Foundation Trust, Nottingham, UK

**Keywords:** attitudes, risk assessment, heuristics biases, sexual crime, sex offender treatment

## Abstract

Attitudes towards individuals with sexual convictions is an area with growing research interest, but the effects of such attitudes on professional judgments is largely unexplored. What is known from the existing literature is that attitudes guide the interpretation of sexual crime related information, which cascade into potential biased or heuristically driven judgments. In this study we recruited samples of both students (*n* = 341) and forensic professionals (*n* = 186) to explore whether attitudes towards individuals with sexual convictions predicted risk judgments of hypothetical sexual offense scenarios, and whether this relationship is moderated by professional status or perpetrator characteristics. Forensic professionals expressed more positive attitudes overall, but the significant effect of attitudes on risk judgments was consistent between participant groups and was not moderated by perpetrator age or sex. We suggest that relying on attitudes as a basis for risk judgments opens the door to incorrect (and potentially dangerous) decision-making and discuss our data in terms of their potential clinical implications. An open-access preprint of this work is available at https://psyarxiv.com/rjt5h/.

Attitudes towards individuals with sexual convictions are important due to their influence on legislation and policies related to the management and sentencing procedures for this group (Harper & Hogue, 2014; Rosselli & Jeglic, 2017), jury decision-making (Wevodau et al., 2016), treatment outcomes (Beech & Hamilton-Giachritsis, 2005), and the social reintegration of individuals upon their release from prison (Göbbels et al., 2012; Harper et al., 2017; Willis et al., 2010). In this study, we explore attitudinal differences towards individuals with sexual convictions among undergraduate students and professionals who work with this population. However, our focus is not simply on describing the same differences that have been reported in previous reviews (Harper et al., 2017; Hogue & Harper, 2019). Instead, we investigate the effects of attitudes on risk judgments made by these groups in relation to hypothetical perpetrators of sexual offenses.

## Attitudes towards Individuals with Sexual Convictions

Sexual crime evokes a strong visceral reaction from the public, and it has been consistently demonstrated that attitudes towards individuals with this conviction type are more negative than those towards expressed towards the perpetrators of other types of criminal offense (Kerr et al., 2018; Olver & Barlow, 2010; Rogers & Ferguson, 2011). This is relevant when considering public support for, and engagement with, community-based interventions that are ostensibly designed to reduce sexual recidivism. For example, there is widespread public support for punitive policies such as community notification and registration (Brown et al., 2008; Salerno et al., 2010; Schiavone & Jeglic, 2009; Shackley et al., 2014; Tewksbury & Mustaine, 2013) despite a lack of empirical evidence that these are effective for reducing reoffending (Levenson et al., 2007, 2010). On the other hand, progressive initiatives such as community-based Circles of Support and Accountability (CoSA) have a relatively strong evidence base (Duwe, 2013; Höing et al., 2013, 2017) but struggle to recruit volunteers to work with individuals with sexual convictions (Höing et al., 2016; Lowe et al., 2019; Richards & McCartan, 2018).

The media (specifically newspapers) has been considered a key source of information about sexual crime, and the origin of many of the psychological processes involved in the formation and expression of attitudes towards the population (Harper & Hogue, 2014, 2015a, 2017; Harper et al., 2017; King & Roberts, 2017; Malinen et al., 2014). According to Harper et al. (2017) there are three key heuristics (i.e., mental shortcuts in decision-making) that guide how people make judgments about sexual crime and the individuals who commit these kinds of offenses. A consideration of heuristics is particularly important when most research instruments designed to explore attitudes related to individuals with sexual convictions uses the general label of “sexual offenders.” As such, our collective understanding of attitudes towards this group is to some extent limited by respondents’ general views about that label, be they guided by cognitive or affective triggers (see Harris & Socia, 2016).

The first major heuristic that promotes sexual crime as a major social issue is that of *availability*. The availability heuristic can be defined as the process by which easily retrievable issues or examples are seen as being more important or prominent than alternatives. For example, when prompted to “name a type of fruit,” most people in Western contexts are likely to lists apples, pears, and bananas over jackfruit or durian due to their more regular encounters with the former examples. In the context of sexual crime, individuals may be increasingly likely to view this as an important social and political issue in times whereby it is covered more in the media, making sexual crime more “available” than other topics (see Harper & Hogue, 2015a, 2017). For example, in the aftermath of the Jimmy Savile scandal in the UK, the British media’s coverage of sexual crime increased by around 300%, even when excluding Savile-related stories from the analysis (Harper & Hogue, 2017). Similarly, the availability of the *#MeToo* campaign has placed sexual harassment higher in people’s minds as a political and social priority for change (Sunstein, 2019).

Relatedly, media coverage may produce a fixed view about who the aforementioned “sexual offenders” are, by only covering certain types of crime. In one of the first major investigations of media coverage about sexual crime, Greer (2003) reported how newspapers tend to report highly sensationalized serial offenses, typically committed by men against children and female strangers (see also Harper & Hogue, 2014, 2017; King & Roberts, 2017; Rogers et al., 2011). This is an example of the development of a *representativeness heuristic*, where judgments about sexual crime become easier to make when an example is closer to the cultural stereotype, and more nuanced when it does not correspond to the stereotypical image. For example, there is an established literature that reports how female-perpetrated sexual offenses are viewed as less serious or harmful than those committed by males, and deserving of a lesser punishment (Clements et al., 2014; Gakhal & Brown, 2011; King & Roberts, 2017; Zack et al., 2018).

The representativeness heuristic can seemingly have profound effects not only on generalized attitudes towards individuals with sexual convictions, but also in relation to how people make attributions of risk about this group. For example, research exploring hypothetical judgments about both adult and juvenile perpetrators of child molestation has demonstrated that people hold more positive attitudes towards a juvenile male with sexual convictions than an adult with similar offending behavior (e.g., Sparks & Wormith, 2021), ascribe less punitive sentences to juvenile-perpetrated crimes (Harper & Bartels, 2017, 2018), and may see juveniles as more amenable to long-term behavioral change (Sahlstrom & Jeglic, 2008). It may therefore be the case that attitudes towards individuals with sexual convictions are based upon how closely the given example matches a “sexual offender schema” (Harper & Bartels, 2018, p. 277) that becomes semantically and affectively entangled with this offense label (Harris & Socia, 2016). From here, the schema activates an attitudinal orientation that guides a range of responses, including sentencing preferences and risk assessments.

## Attitudes within the Professional Context

It is important to explore forensic professionals’ attitudes towards individuals with sexual convictions as their views are likely to influence their practice and thus could have significant clinical implications. Notably, having too positive views may lead to boundary violations, the missing of key case details, and contribute to attributions of lower risk than might be objectively warranted (Blumenthal et al., 2010). Alternatively, negative attitudes can impede the therapeutic relationship, worsen institutional climates conducive to change, and contribute to reduced treatment effectiveness (Beech & Hamilton-Giachritsis, 2005; Craig, 2005; Howard et al., 2019; Marshall et al., 2003; Stasch et al., 2018).

Although professionals working with individuals with sexual convictions appear to hold more negative attitudes towards this group than towards people convicted of other offense types (Craig, 2005; Kjelsberg & Loos, 2008), they have been consistently found to have more positive attitudes than non-forensic professionals, members of the public, and students (Ferguson & Ireland, 2006; Gakhal & Brown, 2011; Harper et al., 2017; Higgins & Ireland, 2009; Hogue, 1993; Hogue & Harper, 2019; Kerr et al., 2018; Kjelsberg & Loos, 2008; Sanghara & Wilson, 2006). However, there is a variation in attitudes between different disciplines, with the degree of specialization driving attitudes. That is, those with the greatest level of therapeutic contact (e.g., psychologists and probation officers) have the more positive attitudes, especially compared to those who are involved in law enforcement processes (Day, 2014; Hogue, 1993; Hogue & Peebles, 1997; Tewksbury & Mustaine, 2013).

It has been proposed that increased contact through experience of working with people with sexual convictions may explain the positive attitudes held by professionals in comparison to the public and student samples (Hogue, 1993; Kerr et al., 2018; Lea et al., 1999; Rosselli & Jeglic, 2017). This is consistent with the representativeness heuristic being a driver of attitudes, with public perceptions being driven by a media-proliferated stereotype and professional attitudes by direct experience (Church et al., 2008; Craig, 2005; Ferguson & Ireland, 2006; Kjelsberg & Loos, 2008; Sanghara & Wilson, 2006; Willis et al., 2010). However, Gakhal and Brown (2011) argue that this effect cannot explain why students would hold more positive attitudes (or, perhaps more accurately, less negative) attitudes than the broader public. On this point, education level may be an important variable. Although there are some studies finding no relationship between education level and attitudes towards individuals with sexual convictions (Nelson et al., 2002; Olver & Barlow, 2010; Payne et al., 2010), this could be questioned due to scoring errors in the scoring of attitudinal scales, small and unrepresentative samples, and the potential over-fitting of data with high numbers of predictors in statistical models. However, a collection of more recent work has reported how a higher level of educational attainment appears to be associated with more positive attitudes towards individuals with sexual convictions (Brown, 1999; Harper et al., 2017; Harper & Hogue, 2015b; Shackley et al., 2014; Willis et al., 2013). Thus, it may be that student attitudes towards this group are driven less by specific views about those who commit sexual offenses, and more by attributions about the potential for behavioral change that come from a more liberal social outlook that tends to be associated with increasing education (Harper & Bartels, 2017). However, this specific mechanism of education leading to exaggerated views about the chances of change among individuals with sexual convictions has not been explored. In this paper, we chose to compare the attitudes of professionals working with individuals with sexual convictions to psychology students who may be in a position to work therapeutically with this population in their future careers. This decision was not designed to act as a proxy for education, but the student sub-sample acts as a contrast group when exploring the effects of attitudes on subsequent risk-related judgments among professionals.

Irrespective of the precise mechanisms of attitudinal formation among forensic professionals working with individuals with sexual convictions, these views could impact processes related to risk assessment. Within the mental health domain, visceral emotional views about service users have been found to be a better indicator of professionals’ assessment of future risk than actuarial case information (e.g., Blumenthal et al., 2010; de Vogel & de Ruiter, 2004). This is evidence of the affect heuristic, with mental health diagnoses triggering an emotional response to the service user, which subsequently determines a judgment of potential risk. In relation to judgments of individuals with sexual convictions, unpublished data from Browne (2017) suggests that more negative attitudes were significantly related to higher estimates of risk among a sample of paraprofessionals who were working or studying within the disciplines of psychology, law, nursing, and teaching. Similarly, Tan (2014) sampled 35 forensic mental health professionals who regularly conduct risk assessments and found a relationship between attitudes towards individuals with sexual convictions and judgments of risk made by professionals. Again, these data are unpublished. Here, more positive attitudes were again associated with lower risk estimates. As such, there is some emerging evidence that the attitudes of forensic professionals can impact on their risk judgments for specific cases. However, the limited sample sizes of much of this work, coupled with fact that these datasets have not been peer-reviewed, prevents us from drawing firm conclusions about the nature of this relationship. As such, in this work we set out to explore the relationship between attitudes and risk judgments about individuals with sexual convictions among professionals in a larger sample than has been previously been studied, and to compare such relationships to a sample of participants with no experience of working with this population.

## The Current Study

As discussed above, the attitudes of professionals working with individuals with sexual convictions may play an important role in their work with this population, which could have profound effects on outcomes related to treatment effectiveness, risk assessments, and parole decisions. For this reason, it is important to first establish the nature of the relationship between generalized attitudes towards individuals with sexual convictions and hypothetical professional practice, before establishing ways of mitigating this link if it is present. In this work our aim is to explore the first part of this problem. In doing so, we look at whether attitudes towards individuals with sexual convictions are predictive of risk judgments about a hypothetical perpetrator of a sexual offense, and whether this relationship is moderated by professional status (i.e., whether it was consistent for professionals and students) and the representativeness of perpetrator characteristics (i.e., if any moderated relationship held for male, female, and juvenile perpetrators). A student comparison group was chosen opportunistically in this study. That is, theoretically we might not expect professionals’ attitudes to be correlated with their risk judgments due to their professional training and experience, but we would expect a relationship in a non-professional sample, such as students. In accordance with these aims, we made three confirmatory hypotheses:

*H1:* Forensic professionals working with individuals with sexual convictions will express significantly more positive attitudes towards this population than students.
*H2:* There will be a significant relationship between attitudes towards individuals with sexual convictions and risk judgments related to hypothetical cases, such that those participants with more negative attitudes will demonstrate perceptions of increased risk.
*H3:* The relationship between attitudes towards individuals with sexual convictions and hypothetical risk judgments will be moderated by professional status, whereby there will be a significant attitude-risk relationship among students but not professionals.

Owing to the complexity of predicting three-way interactions, we sought to explore the effect of perpetrator representativeness on the attitude-risk relationship in a non-confirmatory manner.

## Methods

As authors, we take responsibility for the integrity of the data and the accuracy of the data analyses, and have made every effort to avoid inflating statistically significant results. We also report how we determined our sample size, all data exclusions, all manipulations, and all measures in the study. Further, we have made scored data and all materials available to access at https://osf.io/5fb3y/?view_only=308c156e39f74867b4e3ef1f1bac0ed3. This research was not preregistered.

### Participants

Not knowing the potential sample pool and having a difficult-to-reach professional population as one target sub-sample, we aimed to recruit as many participants as possible to both sub-samples to maximize power, rather than setting out with a specific stopping rule. However, an a priori power analysis using the G*Power application (Faul et al., 2007) suggested that a minimum sample of 199 would be required to detect medium-sized effects with 90% power in regression analyses, and 206 would be required with the same parameters when using ANCOVA.

A total of 595 started the survey containing this study. However, there were no data for the outcome variable for 68 of these, leaving a final sample of 527. Within this number we had two groups of participants. The first was comprised of undergraduate and postgraduate students (*n* = 341)*,* primarily studying psychology courses at the authors’ institution (97%). The remaining 3% of this sample were based in mainland Europe (*n* = 3), North America (*n* = 4), or Australia/New Zealand (*n* = 1). One student participant did not disclose their sex or location. The average age of the student sample was 20.41 years (*SD* = 3.64), with 87% being female. The second group was comprised of professionals who work with individuals with sexual convictions (*n* = 186). Again, most of this group was based in the UK (76%), with sizeable minorities from mainland Europe (*n* = 12), North America (*n* = 23), and Australia/New Zealand (*n* = 3). Six participants did not disclose either their sex or their location. The average age of the professional sample was 41.13 years (*SD* = 12.24), with 86% being female. We had a variety of occupations and working locations represented within the sample, including psychologists and interventions facilitators (64%), social workers (5%), academics (7%), and counselors (4%). The remaining 21% of this sample either did not state their occupation or worked in another role. There were good levels of representation of professionals working in hospitals (22%), prisons (37%), the community (30%), or another context (12%). The average amount of professional experience was 12.50 years (*SD* = 9.10).

We made use of a range of recruitment channels when sourcing participants. Most of our student sample was recruited through an institutional research participation scheme wherein individuals receive course credits following completion of the online questionnaire. To target professionals, we posted the survey link on the LinkedIn page “*Sexual Offender Treatment and Risk Assessment,*” which is a group for individuals who work with or have an interest in individuals with sexual convictions. We also made use of our own personal networks to share the survey link with colleagues and professional contacts. As such, we used opportunity and snowball sampling techniques in our recruitment for this study. No payment was offered, save for institutional research credits for student participants within our own institution.

## Materials

### Demographics

Participants were asked to indicate their gender, age, country of residence, and whether their occupational status was as a student or a professional. Additionally, those who reported that they were a professional were asked to provide their area of work (such as prison, community, or mental health settings), the number of years’ experience they possess in working with people with convictions, and for a description of their current job role.

#### Attitudes to Sexual Offenders Scale (ATS-21; Hogue & Harper, 2019)

The ATS-21 is a shortened version of the original ATS tool (Hogue, 1993) that consists of 21 statements related to individuals with sexual convictions. It has previously demonstrated very good reliability and validity across multiple contexts (see Hogue & Harper, 2019). The measure is comprised of three subscales which capture the three components of attitudes proposed by Breckler (1984), which suggests affective, behavioral, and cognitive processes underpin attitudes towards any given attitudinal target. On the ATS-21, the “Trust” subscale represents the affective component (e.g., “I would like associating with some sex offenders”), the “Social Distance” subscale represents the behavioral component (e.g., “If sex offenders do well in prison/hospital, they should be let out on parole”), and the “Intent” subscale represents the cognitive component (e.g., “Sex offenders only think about themselves”). Consistent with Hogue and Harper’s (2019) suggestions, we used the ATS-21 in a unidimensional way, with participants rating their agreement with each statement on a 5-point scale ranging from 0 (strongly disagree) to 4 (strongly agree). This scoring protocol means that the total score can range from 0–84, with higher scores indicating more positive attitudes towards individuals with sexual convictions. The ATS-21 demonstrated excellent levels of internal consistency (α = 0.94).

#### Case Vignettes

Three sexual offense vignettes were composed for the purpose of this research to facilitate the experimental manipulation. Each vignette was approximately 300 words in length and, consistent with other work in this area, used consistent wording to describe a sexual offense whereby only the experimentally salient details (i.e., representativeness) were changed (Harper & Bartels, 2017, 2018). We were keen to avoid conflating risk judgments with details of a violent contact offense. As such, our vignettes each described the perpetrator grooming a 10-year-old child of the opposite sex over social media whilst posing as a child of a similar age and asking them to perform sexual acts on camera. The perpetrator is depicted as having completed a treatment program in prison. Our experimental factor (representativeness) was divided into three levels, with one vignette for each of these. In one vignette the perpetrator was an adult male (representative), and in the other two were either an adult female (non-representative), or male juvenile (non-representative). Adult perpetrators were labeled as 30-years-old, whereas the juvenile perpetrator was labeled as 16-years-old. The full wording of the vignettes can be found on the project’s OSF page (https://osf.io/5fb3y/?view_only=308c156e39f74867b4e3ef1f1bac0ed3). However, the adult male perpetrator vignette was as follows:

Graham is a 30-year-old male with a sexual interest in pre-pubescent girls. He has never had a long-term relationship before as he lacks confidence to approach women and is not sexually attracted to women his age. He created a fake profile on the social networking site, Facebook, posing as a 13-year-old boy in order to interact with young girls. Whilst using his fake profile, Graham befriended a ten-year-old girl named Sophie. Graham began messaging Sophie, posing as a schoolboy in a nearby school to the one she attends, and they spoke regularly for a period of two weeks. Once he believed he had gained her trust, Graham began sending messages of a sexual nature and attempted to get Sophie to reciprocate. Graham sent multiple sexually explicit messages, and then asked Sophie to send a picture of herself naked. Sophie was reluctant to do this and asked Graham to send one first. Graham took a picture off the internet to send her, and she later agreed to send him a photo of herself. He then escalated his requests, asking for Sophie to go on video and perform sexual acts on herself. Sophie felt uncomfortable with this and told one of her friends, who suggested that Sophie informs the police.

Graham was subsequently arrested and charged with a sexual offence, where he pleaded guilty. Prior to this, he had no previous criminal convictions, but police found hundreds of indecent images of children on his laptop. He was sentenced to 5 years in prison. Whilst serving his sentence, Graham has completed the sex offender treatment programme. Graham says that he has since realised that what he did was wrong, and accepts full responsibility for his actions. He has a parole hearing coming up next month where it will be considered whether he has done sufficient work in prison to warrant release.

#### Risk Judgments with Confidence Rating

Our key outcome variable (risk assessment) was measured using an eight-item scale that was purpose-created for this study. The items were informed by factors used within risk assessments used in forensic practice, including the Violence Risk Scale—Sexual Offender version (Olver et al., 2018). Participants rated each item using a 6-point scale from 0 (strongly disagree) to 5 (strongly agree). We specifically used a scale with an equal number of response options to avoid the potential for participant apathy in choosing a mid-point value (i.e., participants were forced to “disagree” or “agree” with each statement, even if that was to a slight degree. The statements comprising this scale are presented below, with items 4 and 6 being reverse-scored.

1. The individual is likely to commit a further sexual offense.
2. The individual needs to do more treatment to reduce the likelihood of sexual offending.
3. The individual lacks self-control over their urges.
4. The individual poses no danger to the general public.
5. The individual has deviant sexual interests.
6. Release from prison should be recommended for the individual at the parole hearing.
7. The individual is likely to commit a non-sexual offense.
8. The individual poses a danger to children.

The scores from each item were summed to give a composite score ranging from 0-40, with higher scores equating to a higher risk rating (α = 0.81). Additionally, participants were asked to rate their confidence in their risk judgments using one item which could range from 0 (not at all confident) to 5 (extremely confident). We included this confidence score as a covariate in our analysis to control for participant (un)certainty in their opinions.

#### Perceptions of Sex Offenders Scale (PSO; Harper & Hogue, 2015a)

The PSO is a revised version of the Community Attitudes to Sex Offenders (CATSO) scale (Church et al., 2008) produced by Harper and Hogue (2015b) after concerns about the CATSO’s theoretical validity. The scale consists of 20 statements pertaining to respondents’ views about “Sentencing and Management” of individuals with sexual convictions (e.g., “People who commit sex offences should be subject to harsh restrictions on their liberty for the rest of their lives”; α = 0.92), “Stereotype Endorsement” (e.g., “Most sex offenders do not have close friends”; α = 0.84), and “Risk Perception” (e.g., “People are far too on edge about the risks posed by sex offenders”; α = 0.72). Each item is framed as a statement, against which participants rated their level of agreement using a 6-point scale anchored from 0 (strongly disagree) to 5 (strongly agree). Total scores for each subscale were computed, with higher scores indicating more punitive views, greater endorsement of stereotypes, and increased risk perceptions, respectively.

### Procedure

Ethical approval was gained from the Nottingham Trent University School of Social Sciences, Research Ethics Committee prior to data collection. The present study was conducted using an online survey hosted by *Qualtrics* to allow for remote access and anonymous participation, with the link being distributed in the places described above. Those who were interested in taking part could click the link to receive more information about the research. There was no deception in this information, though the full purpose and aims of the research were not disclosed to reduce demand characteristics. If participants opted to proceed with the study, they were then presented with the demographic questions, before completing the ATS-21 to measure their baseline attitudinal orientation. Participants were then randomly assigned by the survey software to one of the three experimental vignettes. After reading their vignette, participants were asked to complete the risk judgments measure and indicate their confidence in their risk ratings. The PSO was then presented at the end of the survey before participants were fully debriefed on the purpose and hypotheses of the study. To aid replication, an anonymized version of the Qualtrics survey in .qsf format is available at https://osf.io/5fb3y/?view_only=308c156e39f74867b4e3ef1f1bac0ed3.

## Results

### H1: Group Differences in Attitudes

We used a series of between-subjects two-way analyses of variance (ANOVAs) to examine attitudes towards individuals with sexual convictions, mean risk judgments, confidence in risk judgments, and perceptions of individuals with sexual convictions. Descriptive statistics are presented in Table 1.

**Table 1.** Descriptive Statistics for Attitudes, Risk Judgments, Judgment Confidence Ratings, and PSO Scores, by Group.

	Students			Professionals		
	*Male*	*Female*	*Juvenile*	*Male*	*Female*	*Juvenile*
*ATS-21*	44.62 (12.42)	42.00 (11.94)	42.98 (12.01)	59.24 (10.20)	59.84 (9.07)	59.73 (8.93)
*Risk rating*	25.32 (5.94)	24.78 (5.62)	23.70 (6.04)	21.59 (5.90)	19.44 (4.86)	18.73 (5.39)
*Confidence*	2.85 (1.10)	2.84 (1.09)	2.82 (1.16)	3.35 (1.15)	3.56 (1.28)	3.39 (1.23)
*PSO sentencing*	16.86 (9.49)	17.70 (8.35)	15.59 (8.25)	7.15 (6.41)	6.33 (4.16)	6.90 (5.42)
*PSO stereotypes*	12.07 (4.39)	10.60 (4.17)	10.32 (4.25)	9.45 (4.77)	8.47 (4.07)	9.36 (4.09)
*PSO risk*	45.87 (3.92)	16.05 (3.74)	16.16 (3.65)	14.19 (4.86)	12.43 (3.93)	13.34 (4.52)

*Note.* Data represent mean values with standard deviations presented in parentheses.

In the first analysis, we ran a 2 (Group) × 3 (Vignette) ANOVA on ATS-21 scores. This allowed us to test H1 (i.e., that professionals would express more positive attitudes than students) while simultaneously checking for consistency in baseline attitudes between participants across our experimental conditions. We found a significant main effect of group membership (*F*(1, 521) = 255.92, *p* < .001, η_p_^2^ = 0.33), whereby professionals expressed more positive attitudes than students (*M*_diff_ = 16.40, *p* < .001, *d* = 1.52). There was no main effect of vignette (*F*(2, 521) = 0.33, *p* = .722, η_p_^2^ < 0.01), nor was there an interaction between these variables (*F*(2, 521) = 0.85, *p* = .426, η_p_^2^ < 0.01). Collectively, these findings replicate past research showing more positive attitudes towards individuals with sexual convictions among professionals (supporting H1) but show that baseline attitudes did not systematically differ between participants randomly assigned to each experimental vignette.

We then ran the same 2 × 3 ANOVA separately for both risk judgment outcomes and confidence ratings. In relation to risk judgments, we found a significant main effect of group membership (*F*(1, 512) = 79.60, *p* < .001, η_p_^2^ = 0.14), whereby professionals expressed judgments equating to lower risk across the vignettes than did students (*M*_diff_ = −4.68, *p* < .001, *d* = −0.81). There was also a significant main effect of vignette (*F*(2, 512) = 6.17, *p* = .002, η_p_^2^ = 0.02), whereby the adult male case was ascribed significantly more risk than the juvenile perpetrator case (*M*_diff_ = 2.24, *p* = .002, *d* = 0.32). The adult female case sat between these extremes but did not differ significantly from either of them. There was no interaction between these two variables, *F*(2, 512) = 0.86, *p* = .426, η_p_^2^ < 0.01.

When examining confidence in risk judgments, we found a significant main effect of group membership (*F*(1, 520) = 14.96, *p* < .001, η_p_^2^ = 0.03), whereby professionals were more confident in their judgments than students (*M*_diff_ = 0.41, *p* < .001, *d* = 0.35). However, there was no effect of vignette (*F*(2, 520) = 0.37, *p* = .693, η_p_^2^ < 0.01), nor an interaction between the two variables (*F*(2, 520) = 0.38, *p* = .697, η_p_^2^ < 0.01). These data suggest that increased professional confidence was consistent across all vignette conditions.

On the PSO we considered each subscale individually. In relation to “Sentencing and Management,” there was a significant main effect of group membership (*F*(1, 509) = 188.85, *p* < .001, η_p_^2^ = 0.27), whereby professionals were less punitive than students (*M*_diff_ = −14.53, *p* < .001, *d* = −1.37). However, there was no main effect of vignette (*F*(2, 509) = 0.51, *p* = .600, η_p_^2^ < 0.01), nor an interaction between the two variables (*F*(2, 509) = 1.19, *p* = .305, η_p_^2^ = 0.01).

In relation to “Stereotype Endorsement,” there was a significant main effect of group membership (*F*(1, 509) = 194.25, *p* < .001, η_p_^2^ = 0.28), whereby professionals endorsed fewer stereotypes than students (*M*_diff_ = −1.90, *p* < .001, *d* = −0.44). There was also a significant main effect of vignette (*F*(2, 509) = 3.47, *p* = .032, η_p_^2^ = 0.01), whereby participants presented with the adult male case endorsed significantly more stereotypical thinking than those presented with the adult female case (*M*_diff_ = 1.23, *p* = .035, *d* = 0.28). The juvenile case sat between these extremes in terms of the level of stereotypical thinking it elicited but did not differ significantly from either of them. There was no interaction between the two variables (*F*(2, 509) = 1.19, *p* = .305, η_p_^2^ = 0.01), meaning that the effect of the vignettes on stereotype endorsement was consistent in both groups.

In relation to “Risk Perception,” there was a significant main effect of group membership (*F*(1, 509) = 52.94, *p* < .001, η_p_^2^ = 0.09), whereby professionals perceived less risk than students (*M*_diff_ = −2.70, *p* < .001, *d* = −0.65). However, there was no main effect of vignette (*F*(2, 509) = 1.56, *p* = .211, η_p_^2^ < 0.01), nor an interaction between the two variables (*F*(2, 509) = 2.29, *p* = .102, η_p_^2^ = 0.01).

### H2 and H3: Effects of Attitudes on Risk Judgments

To explore the relationships between our variables we first ran a correlational analysis. As expected, the strongest relationships were relevant to ATS-21 scores, with large correlations between attitudes and case risk judgments, punitive sentencing and management preferences, and risk perceptions on the PSO. Risk judgments were correlated to a minor degree with judgment confidence. Increasing age also had significant relationships with more positive attitudes, lower risk judgments (both in relation to the presented case and globally as assessed using the PSO), and less punitive sentencing and management preferences. Our self-created risk judgment scale was significantly correlated with the PSO’s “Risk Perception” subscale, providing further evidence of its construct validity. All correlation coefficients are presented in Table 2.

**Table 2.** Zero-Order Correlations between Measured Variables.

	1	2	3	4	5	6	7	8	9
1. Sex	—								
2. Age	.31^***^	—							
3. Experience (years)	.15	.72^***^	—						
4. ATS-21	.15^**^	.49^***^	.03	—					
5. Risk rating	−.10^*^	−.34^***^	−.08	−.67^***^	—				
6. Confidence	.02	−.17^***^	−.08	−.24^***^	.21^***^	—			
7. PSO sentencing	−.13^**^	−.44^***^	.05	−.84^***^	.67^***^	.22^***^	—		
8. PSO stereotypes	−.03	−.19^***^	−.05	−.23^***^	.23^***^	.05	.28^***^	—	
9. PSO risk	−.17^***^	−.30^***^	−.11	−.61^***^	.53^***^	.11^*^	.52^***^	−.04	—

^*^ *p* < .05, ^**^ *p* < .01, ^***^ *p* < .001.

We used the PROCESS macro for SPSS (Hayes, 2017) to run a moderated moderation analysis. In essence, this is an analysis that looks for main effects and both two- and three-way interactions within a regression model. Our focal predictor (*X*) was participants’ ATS-21 score, which we used to predict the outcome (*Y*) of risk judgments. We then added group (student vs. professional) as the first moderator (*W*) and vignette type (adult male vs. adult female vs. juvenile) as the second moderator (*Z*). To control for participants’ confidence in their judgments, we also entered this score as a covariate (though the removal of this covariate did not significantly change the results). We calculated 95% confidence intervals for regression estimates using 5000 bootstrapped re-samples of the data. All Beta (*B*) coefficients are unstandardized in accordance with Hayes’ (2017) recommendations for using the PROCESS macro.

The model was significant and accounted for slightly less than 50% of the variance in risk judgments (*R*^*2*^= .480, *F*(8, 508) = 58.60, *p* < .001). ATS-21 scores were significantly and negatively related to risk ratings (*B* = −0.33, *t*(509) = −15.75, *p* < .001), with more positive attitudes related to judgments of lower risk. This result is consistent with H2. Group membership was not a significant predictor of risk judgments (*B* = −0.04, *t*(509) = −0.07, *p* = .942), suggesting that students and professionals provided similar estimates of the risk posed by those individuals depicted within the vignettes. Importantly, the interaction between ATS-21 scores and group membership was not statistically significant (*B* = 0.08, *t*(508) = 1.93, *p* = .054). This means that the relationship between attitudes towards individuals with sexual convictions and risk judgments of the hypothetical vignettes was consistent in both students and forensic professionals, contrary to H3. A summary of these findings is presented in Table 3.

**Table 3.** Moderated Moderation Model Coefficients Predicting Risk Judgments from ATS-21 Scores, Group Membership, and Vignette Type.

	*B*	*SE*	*t*	*p*	95% CI (*B*)
*Constant*	*21.98*	*0.71*	*30.90*	*< .001*	*[20.58, 23.38]*
*ATS-21*	−0.33	0.02	−15.75	<.001	[−0.37, −0.29]
*Group*	−0.04	0.58	−0.07	.942	[−1.17, 1.09]
*ATS-21 × group*	0.08	0.04	1.93	.054	[−0.00, 0.16]
*Vignette*	−1.02	0.33	−3.11	.002	[−1.67, −0.37]
*ATS-21 × vignette*	0.01	0.03	0.54	.592	[−0.04, 0.06]
*Group × vignette*	−0.82	0.69	−1.20	.231	[−2.18, 0.53]
*ATS-21 × group × vignette*	0.04	0.75	0.75	.456	[−0.06, 0.13]
*Confidence (covariate)*	0.19	1.06	1.06	.291	[−0.16, 0.54]

**Figure 1.**
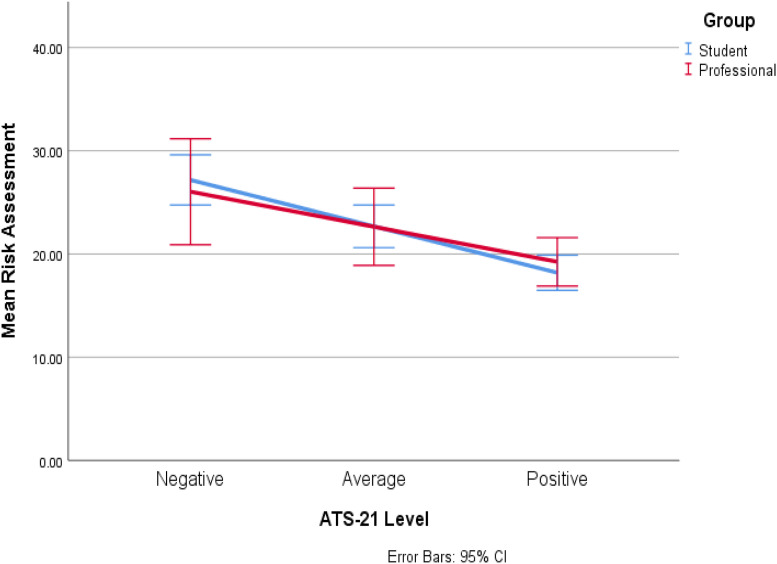
Relationship between ATS-21 scores and risk judgments, by group membership.

Figure 1 visually shows the lack of significant interaction between ATS-21 scores and participants’ group membership when predicting risk judgments. That is, the relationship between ATS-21 scores and risk judgments is consistent between the groups.

As suggested earlier, we examined the vignette-level data in an exploratory manner to see whether the representativeness of the case demographics further moderated the effects of ATS-21 scores on risk judgments. Within the regression model, we observed a significant effect of the vignette condition on risk judgments (*B* = −1.02, *t*(508) = −3.11, *p* = .002). Exploring the mean values for each risk judgment, we can see that this effect is driven by higher ratings of risk assigned to the adult male perpetrator case. The two-way interaction between vignette condition and group membership was not statistically significant, meaning that these differences were consistent in both participant groups. Similarly, there were no significant interactions that involved ATS-21 scores, meaning that the vignette representativeness did not affect the relationship between ATS-21 scores and risk judgments.

## Discussion

In this study we investigated whether there was a relationship between attitudes towards individuals with sexual convictions and hypothetical risk judgments made by both students and forensic professionals who work with this population.

### Interpretation of Key Findings

Consistent with previous research (Ferguson & Ireland, 2006; Gakhal & Brown, 2011; Higgins & Ireland, 2009; Hogue, 1993; Kerr et al., 2018; Kjelsberg & Loos, 2008), the professionals in our sample scored higher on the ATS-21 measure than did students, indicating more positive attitudes towards individuals with sexual convictions among those with a professional background. This was consistent with our expectations (H1). At the average level, professionals were also less likely to rate their assigned case as a risk of reoffending and were more likely to have confidence in their opinion than were students. Relatedly, they were less likely to endorse punitive policy proposals, engage in stereotypical thinking, or infer risk when assessed using the PSO. Collectively, these data indicate that professionals are more positive (or, perhaps more accurately, less prone to stereotypical thinking) about individuals with sexual convictions. This is perhaps a demonstration of the effectiveness of staff training processes that are currently in place for professionals working with individuals with sexual convictions. It could equally be a sign that those with a more open mind about this group are joining the workforce in the first place. Nonetheless, working to improve personal attitudes among forensic professionals who work with individuals with sexual convictions is important because of their potential clinical implications (Harper et al., 2017). Professionals’ attitudes can impact on how they work with service users during treatment (Craig, 2005; Gibson, 2021), and so holding more positive attitudes can help professionals to cultivate positive therapeutic environments that are conducive to more effective treatment and the reduction of dynamic risk factors (Beech & Hamilton-Giachritsis, 2005; Howard et al., 2019; Marshall et al., 2003; Stasch et al., 2018). Whether attitudes are related to actual professional judgments (rather than artificial judgments of hypothetical cases, as in this study) is still an unanswered question, and further empirical work is to establish both whether such a relationship exists, and whether any relationship is positive or negative. However, thinking towards possible interventions to improve attitudes among forensic staff (should this be necessary), there are some brief interventions with reported short-term effectiveness (Craig, 2005; Hogue & Peebles, 1997). A positive step to incorporate such packages into core forensic psychology training among students enrolled on entry level courses (e.g., forensic psychology undergraduate programs) to ensure that these future professionals enter the field with an appropriate attitudinal outlook for working with this population.

Consistent with H2, we observed a significant negative relationship across all participants between ATS-21 scores and risk judgments of a hypothetical sexual offense case. That is, the more negative participants were about this population, the higher they estimated the risk level of an individual with a sexual conviction presented in a hypothetical case vignette. However, contrary to H3, we found no evidence that this relationship was moderated by group membership. This means that the attitudes-risk judgment relationship was the same in both students and professionals who work with individuals with sexual convictions. We believe this result to be our key contribution in this work. That is, although the difference in generalized attitudinal scores between professionals and students is to be expected due to the former group’s experiences of working with this population, but we were surprised (for the same reasons) to see attitudes still having a significant effect on hypothetical professional judgments. This finding should be of great concern when considering the potential effects of attitudes on professionals’ ability to produce objective and accurate risk assessments. Such judgments have a significant influence in decision-making, including those decisions made about parole (Blumenthal et al., 2010; Harper et al., 2017). Inaccurate risk assessments that over- or under-estimate risk (in the case of more negative or more positive assessor attitudes, respectively) could lead to either the unnecessary deprivation of liberty, or the release of potentially dangerous individuals back into the community. As such, there is a broad appreciation that professional risk assessment outcomes should be independent of assessor bias. However, research has found that professionals believe that they are able to conduct objective assessments and to minimize the potential impact of their own bias, but are able to recognize bias in others (Neal et al., 2018; Zapf et al., 2018). This indicates that forensic professionals may require more awareness into the presence and potential impact of their own attitudes for influencing their professional decision-making. As such, it is incumbent upon criminal justice institutions and structures to produce a context within which risk assessments can be conducted in a way that is relatively free from personal assessor attitudes and biases.

The finding that the relationship between ATS-21 scores and risk judgments was not moderated by the age or sex of the individual being assessed is interesting. Previous work has found that these variables do appear to play a role in the expression of attitudes to individuals with sexual convictions, and preferences for punishment over rehabilitation (Gakhal & Brown, 2011; Harper & Bartels, 2017, 2018; Higgins & Ireland, 2009; Sparks & Wormith, 2021). It is not our contention that these findings are wrong, in that the data presented in this paper are less related to absolute levels of positivity or negativity toward different perpetrator demographics, and more related to their effect on the attitude-judgment relationship. That is, although past research does find that, at the raw judgment level, judgments of different “types” of individuals with sexual convictions do seem to differ, these perpetrator characteristics do not alter the independent relationship between generalized attitudes and risk judgments. This again highlights the pervasive nature of attitudes toward individuals with sexual convictions, in that important perpetrator characteristics do not alter their effects on professional judgments.

These data point towards the importance of not relying only on professional clinical judgment and more structured assessments of risk into forensic practice. The combination of risk assessment methods (i.e., the inclusion of actuarial methods to assess risk) has been suggested for some time, but the findings reported here provide some preliminary evidence as to why professional clinical judgments of risk may underperform when predicting future offending as compared to structured alternatives (Hanson & Morton-Bourgon, 2009; Helmus & Bourgon, 2011; Hilterman et al., 2014; Ægisdóttir et al., 2006; Dawes et al., 1989). According to Helmus (2018), the use of actuarial measures—those which specifically and objectively predict recidivism using a small number of fixed factors known to be associated with future offending—offers a reliable and transparent method of decision-making about risk in accordance with the risk principle of the risk-need-responsivity model (Andrews et al., 1990, 2011; Lovins et al., 2009; Smid et al., 2013). As such, using such structured assessments potentially offsets the effects of assessor attitudes that may cloud more unstructured methods.

### Limitations and Future Directions

One limitation of this research was the use of explicit self-report measures to assess the sensitive and politically charged topic of attitudes towards people with sexual convictions which could lead to socially desirable responding (Harper et al., 2017). However, recently published data from Hogue and Harper (2019) found that ATS-21 scores were uncorrelated to scores on a social desirability scale, suggesting that the ATS-21 can provide an accurate insight into individuals’ attitudes towards people with sexual convictions. Future research could look to replicate our findings using indirect measures of attitudes, such as a single-target implicit association test (IAT). Previous work using such tools has reported a significant correlation of moderate effect size (*r* = 0.41) between explicit and implicit attitudes towards individuals with sexual convictions (Malinen et al., 2014), which suggests a relationship (though not a perfect concordance) between attitudes expressed both consciously and automatically.

Related to sampling, and noting the educational effect on attitudes towards individuals with sexual convictions, it may have been prudent to include a sensitivity analysis in comparing undergraduate and graduate students in our sample. In this work We only asked participants if they were a “student” or “professional,” and so cannot conduct such an analysis. Replications might look to investigate such differences based on the specific educational levels of student controls. Similarly, our observed relationships between age and attitudes and risk perceptions might have been driven by the more positive attitudes of the professional sample, due to the relatively homogenous nature of the student controls. That is, perhaps this represents more an artificial correlation than one that truly reflects a relationship with age. Community replications would be a useful step to establishing whether age is actually correlated with such outcomes. We also acknowledge that our samples are “WEIRD” in nature (see Henrich et al., 2010) and cross-cultural collaborative replications should be conducted to test the generalizability of our findings.

We used purpose-written hypothetical case vignettes alongside an artificial risk judgment measure based on a risk assessment tool than many professionals may be unfamiliar with. We believe that this was a good way to initially investigate the theoretical link between attitudes and risk judgments while using an international sample, as each country appears to have its own set of norms in relation to specific risk assessment instruments and risk level assignment (for a discussion of risk classifications and the need for a common risk language, see Hanson et al., 2017). However, these choices do mean that our findings lack a degree of ecological validity (and indeed diversity, with us using cisgendered perpetrators involved in heterosexually-framed offending). Future work might look to measure professional attitudes independently, and then look to explore the predictive validity of these in relation to actual risk assessments that have been conducted. Relevant outcomes might include risk categorization, parole recommendations, and the linguistic composition of risk assessment reports. Similarly, our recruitment of participants was not limited to any particular country or working context, which may have introduced cross-cultural variations which was not controlled for. Furthermore, the findings from the present study may not be generalizable due to the high proportion of female participants which may not be representative of all student and forensic professional populations. Future work should look to explore country-, tool-, and assessor-specific issues that might influence the strength of the attitude-risk judgment relationship.

Finally, we used a very specific type of sexual offense in our vignette. Arguably, this unrepresentative offense type (based on media coverage; Harper & Hogue, 2015a, 2017) should have lessened the effect of attitudes on risk judgments. However, future research is needed to examine whether this relationship holds for other types of sexual offense, and if attitudes have similar influences of risk judgments of people convicted of non-sexual offenses.

## Conclusions

In this work we have demonstrated the pervasive effects of attitudes towards individuals with sexual convictions on risk judgments made by both students and forensic professionals who work with this population. Although we expected an effect such as this among students, it is surprising and worrying to also observe it among those with a professional responsibility for accurately assessing risk in forensic settings. As such, we argue that greater staff training be promoted, and clinical judgments be embedded within structured risk assessment processes, to reduce the potential effects of attitudes on professional judgments of risk. In doing so, we hope that the current data shed light on the importance of not only considering the accurate measurement of valid risk and protective factors within the assessment process, but also in considering assessor-level attitudes and psychological processes to ensure fair and accurate determinations of risk are made.
